# Incidence, risk factors and outcomes of refeeding syndrome in critically ill patients receiving enteral nutrition: a prospective observational study

**DOI:** 10.3389/fmed.2026.1923682

**Published:** 2026-08-31

**Authors:** Linfang Li, Yihui Shen, Na Zhu, Jie Yu, Rongrong Ma

**Affiliations:** 1 Department of Emergency, Affiliated Hospital of Jiangnan University, Wuxi, Jiangsu, China; 2 Department of Emergency, Jiangnan University Medical Center, Wuxi, Jiangsu, China

**Keywords:** critically ill patients, enteral nutrition, mortality, refeeding syndrome, risk factors

## Abstract

**Objective:**

Refeeding syndrome (RFS) is a serious metabolic complication that may increase mortality in patients receiving enteral nutrition (EN). This study aims to investigate the incidence, risk factors of RFS within the ASPEN-defined 5-days window in critically ill patients receiving EN, and to evaluate its association with prolonged ICU length of stay and ICU mortality.

**Methods:**

A single-center prospective observational study was conducted in the emergency intensive care unit. Electrolyte and biochemical analysis were collected at admission and on days 3 and 5 of EN. Data on ICU length of stay and ICU mortality were collected. Univariate and multivariate Firth’s penalized logistic regression analyses were used to identify risk factors for RFS.

**Results:**

A total of 294 patients were enrolled. The incidence of RFS was 62.9% (185/294) on day 3 and 71.4% (210/294) on day 5 of EN (*P* < 0.001). Overall ICU mortality was 12.9% (38/294). Moderate/severe RFS was associated with significantly higher ICU mortality compared with the combined mild/no-RFS group on day 3 (OR = 2.14, 95% CI: 1.06–4.28, *P* = 0.036) and day 5 (OR = 2.47, 95% CI: 1.22–4.99, *P* = 0.014). Multivariate analyses identified baseline serum magnesium, phosphate, and potassium as independent risk factors of RFS.

**Conclusion:**

Refeeding syndrome incidence in critically ill patients receiving EN remains high, with progressive elevation during the first 5 days. Patients who developed moderate/severe RFS on days 3 and 5 had significantly higher ICU mortality than those mild/no-RFS patients. Baseline serum phosphorus, potassium, and magnesium were risk factors of RFS.

## 1 Introduction

Refeeding syndrome (RFS) is a potentially life-threatening metabolic disorder that occurs following the reintroduction of nutrition after a period of nutritional deprivation, with hypophosphatemia being a prominent characteristic (1–3). In critically ill patients, RFS may lead to acute multi-organ dysfunction, significantly prolong hospital length of stay, and independently elevate mortality risk (4–6). Almost all patients in the ICU receive nutritional support, including enteral nutrition (EN) and/or parenteral nutrition (7). Current evidence indicates that nutritional support, particularly EN, is associated with the occurrence of RFS (8–10). Furthermore, critically ill patients commonly experience prolonged negative nitrogen balance, which is compounded by malnutrition-induced intestinal barrier damage, reduced visceral perfusion, and an increased risk of bacterial translocation. These disturbances significantly increase their vulnerability to RFS (11, 12).

The mechanism of RFS involves early refeeding-induced hyperinsulinemia, which accelerates the intracellular shift of phosphate, potassium, magnesium, vitamins and trace elements, leading to precipitous serum depletion. Complications of this syndrome include electrolyte abnormalities (hypophosphatemia, hypokalemia, and hypomagnesemia) along with sodium and fluid retention, potentially leading to heart failure, respiratory failure, or death (13). Additionally, thiamine and micronutrient depletion may cause further end-organ injury. Multi-system involvement frequently results in prolonged mechanical ventilation, increased infection risk, prolonged length of stay, increased healthcare burden and delayed recovery. Delayed recognition and inadequate management of RFS substantially increase the risk of metabolic derangements, consequently affecting the treatment and recovery of critically ill patients (5). A prospective observational cohort study conducted in Malaysia revealed that nearly half of ICU admissions developed refeeding hypophosphatemia (RH), which was commonly associated with concurrent imbalances in phosphate, magnesium, potassium and calcium (14). Another study conducted in Japan involved 955 patients, of whom 33.1%, 26.7%, 37.8%, and 2.4% were classified into the no-risk, low-risk, high-risk, and very high-risk groups, respectively. Corresponding 30-days in-hospital mortality rates were 4.4%, 5.5%, 5.0% and 21.7%, respectively (*p* = 0.047) (15). Earlier work by the same group reported that 48.5% of critically ill patients requiring ICU admission were classified as high or very high risk for developing RFS (16). These data underscore that critically ill patients demonstrate a higher risk of developing RFS. RFS may develop as a complication of nutritional rehabilitation, leading to severe complications and becoming potentially life-threatening, if not recognized in a timely manner and treated adequately (17).

Reported incidence of RFS vary significantly across different studies (18, 19). This heterogeneity stems primarily from inconsistent diagnostic criteria and baseline differences among study populations (19, 20). The pathophysiology of RFS remains incompletely elucidated, and no internationally recognized diagnostic “gold standard” has been established. Consequently, clinical diagnosis mainly relies on a composite assessment of characteristic laboratory indexes and clinical signs. A large retrospective study published in 2023 that focused on 2123 ICU patients confirmed RFS prevalence rates ranging from 1.5% to 88.0% depending on the definition applied (20). The American Society for Parenteral and Enteral Nutrition (ASPEN) defines RFS as an abnormal decline in serum phosphorus, potassium, and/or magnesium levels following the initiation of nutritional support (21). In contrast, Doig et al. used a more restrictive single-parameter criterion: a serum phosphate concentration < 0.65 mmol/L within 72 h after nutritional support (22).

Moreover, additional diagnostic criteria proposed by various researchers continue to appear, further complicating cross-study comparisons of RFS epidemiology. Individuals deemed high risk should undergo dynamic monitoring protocols with serum phosphate as the key target parameter. Nonetheless, current clinical practice reveals a marked under-recognition of RFS; its nonspecific clinical manifestations further contribute to missed diagnoses and diagnostic delays. According to surveys, the majority of RFS occur within the first 72 h after the initiation of nutritional support. Therefore, early and systematic biochemical monitoring is widely recognized as a cornerstone for timely recognition and prevention (1, 23). The risk-stratification criteria published by the UK National Institute for Health and Care Excellence (NICE) incorporate readily quantifiable variables, including body-mass index (BMI), recent weight loss, pre-refeeding electrolyte derangements and so on (23). For patients who may be at risk of developing refeeding syndrome, their serum phosphate levels should be monitored dynamically (2).

Despite the substantial clinical burden of RFS in critically ill patients receiving EN, robust prospective data on its incidence within standardized diagnostic windows remain limited. Most existing studies employ heterogeneous criteria and observation periods, precluding meaningful cross-study comparisons and hindering the development of evidence-based prevention strategies. This study aims to address these gaps by applying the ASPEN 2020 consensus criteria within a strictly defined 5-days observation window and by constructing prediction models that explicitly account for the circularity problem.

In this study, we aim to investigate the incidence and risk factors of RFS in critically ill patients receiving EN. Additionally, we evaluate whether RFS is associated with prolonged ICU length of stay and increased ICU mortality.

## 2 Materials and methods

### 2.1 Study design and ethics

This single-center prospective observational study was conducted in the emergency intensive care unit (EICU) of Hospital between December 1, 2024, and September 30, 2025. A convenience sampling method was used to recruit participants. The study was approved by the Ethics Committee of Hospital (Approval No.: LS2024577). Written informed consent was obtained from each patient or their legally authorized representative prior to enrollment. The study was conducted in accordance with the Declaration of Helsinki and reported following the Strengthening the Reporting of Observational Studies in Epidemiology (STROBE) guidelines.

### 2.2 Participants

Consecutive critically ill patients who received EN in the EICU of Affiliated Hospital of Jiangnan University between December 1, 2024, and September 30, 2025, were screened for eligibility.

#### 2.2.1 Inclusion criteria

(a) Age ≥ 18 years; (b) receiving EN through a nasogastric tube or jejunal tube within 48 h after admission; (c) receiving EN for ≥72 h; (d) the patient or their legally authorized representative provided written informed consent and was willing to participate in this study.

#### 2.2.2 Exclusion criteria

(a) Missing data precluding RFS assessment; (b) interruption or change of EN due to disease progression or treatment-related reasons; (c) hypophosphatemia attributable to other causes, including recent hemodialysis, treatment for hyperphosphatemia, or parathyroidectomy.

### 2.3 RFS definition

In this study, RFS was diagnosed using the criteria published by ASPEN. The ASPEN criteria are as the follows: a decrease in any 1, 2, or 3 of serum phosphorus, potassium, and/or magnesium levels by 10.0%–20.0% (mild RFS), 20.0%–30.0% (moderate RFS), or >30.0% and/or organ dysfunction resulting from a decrease in any of these and/or due to thiamine deficiency (severe RFS). And occurring within 5 days of reinitiating or substantially increasing energy provision.

### 2.4 EN and electrolyte management

During the study period, EN and electrolyte management were implemented according to routine protocols of our center without study intervention. EN was initiated within 24∼48 h of ICU admission for patients who were hemodynamically stable, had functional gastrointestinal tracts, and had no contraindications. Nutritional formula was continuously administered through a nasogastric or nasoenteric tube using an infusion pump, typically at an initial rate of 20∼30 mL/h. The feeding rate was subsequently individualized according to gastrointestinal tolerance, hemodynamic status, and achievement of the prescribed nutritional target. EN was generally administered for 18∼20 h per day, with a feeding-free interval of 4∼6 h. The infusion rate or volume was adjusted according to patient’s clinical situation. Prophylactic supplementation of phosphorus, potassium, or magnesium was not routinely administered. When an electrolyte abnormality was confirmed by laboratory testing, the treating physician determined whether electrolyte replacement was required according to the patient’s clinical condition. An RFS diagnosis did not trigger protocolized nutritional reduction or standardized electrolyte supplementation.

### 2.5 Data collection

Data collection was performed in three stages. First, within the 48 h of admission, patients’ demographics and laboratory test results upon admission were extracted from the electronic medical record system (all patients underwent a standard blood tests upon admission). Baseline information included gender, age, admission time, admission diagnosis, comorbidities, height and weight. Laboratory tests conducted at admission included albumin, globulin, urea, creatinine, uric acid, serum sodium, lactate dehydrogenase, creatine kinase (CK), hemoglobin, blood glucose, serum sodium, potassium, chlorine, magnesium, calcium, phosphorus and C-reactive protein (CRP). Nutritional risk at ICU admission was assessed using the modified Nutrition Risk in the Critically ill (mNUTRIC) score. The score was calculated from age, APACHE II score, SOFA score, number of comorbidities, and the interval between hospital admission and ICU admission. Second, fasting venous blood samples were collected on the day 3 and 5 of EN for electrolyte and biochemical analysis to evaluate in electrolyte levels. Finally, data on ICU length of stay and ICU mortality were collected. The laboratory indices obtained at admission served as the baseline reference levels for patients prior to the initiation of EN. Values on the day 3 and 5 were compared against baseline, respectively. All samples were collected at 6:00 AM prior to the initiation of EN on the day 3 and 5 for electrolyte and biochemical analysis, and were processed within 2 h of collection.

### 2.6 Outcome

Primary outcomes: incidence of RFS on days 3 and 5 of EN, classified by severity (mild, moderate, severe) according to the ASPEN criteria. Secondary outcomes: ICU mortality and ICU length of stay.

### 2.7 Data analyses

Descriptive statistics were calculated for all variables. Continuous variables were presented as means ± SD or medians (IQR) and were dichotomized or categorized according to prespecified clinically reference ranges when necessary. Between-group comparisons were conducted using the independent-samples *t*-test or Mann-Whitney U test for continuous variables and the Pearson chi-square test or Fisher’s exact test for categorical variables. Univariable analyses were performed to identify factors associated with RFS. Variables with *P* < 0.050 were included in multivariable Firth’s penalized logistic regression because of sparse outcome counts and complete or quasi-complete separation. Adjusted ORs with 95% profile penalized likelihood CIs were reported. Conventional logistic regression results were provided in Supplementary Table 1. Mortality rates were compared using the Pearson chi-square test, and trends across ordered categories were assessed using the Cochran-Armitage trend test. Pairwise comparisons were conducted using Fisher’s exact tests. ROC curve analysis was performed to calculate the AUC. All tests were two-sided, with *P* < 0.050 considered statistically significant. Analyses were conducted using SPSS Statistics, version 26.0, and Firth regression was performed in R software.

## 3 Results

### 3.1 Patient characteristics

**FIGURE 1:**
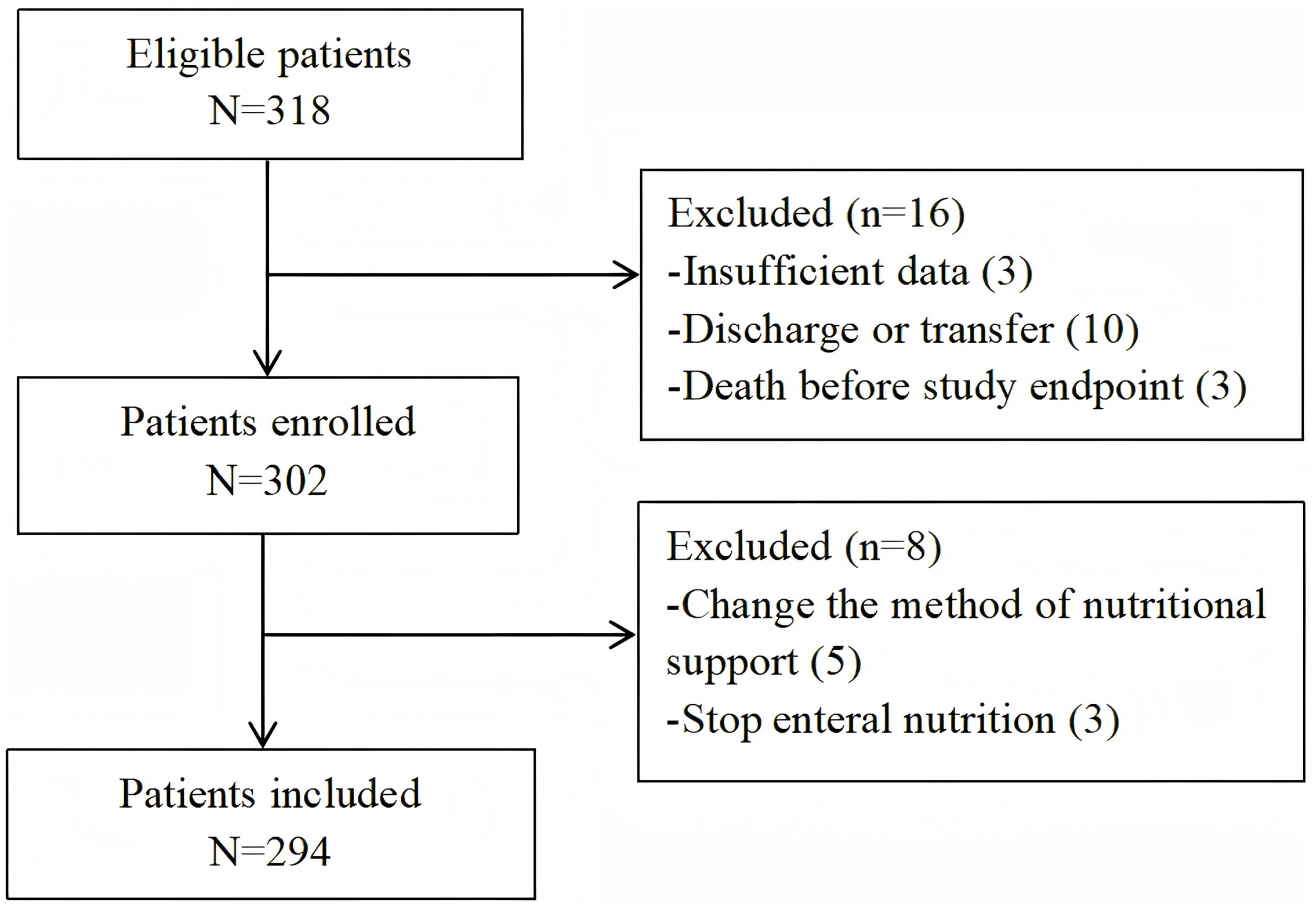
Flowchart of study participant inclusion.

A total of 318 patients were assessed for eligibility based on the inclusion and exclusion criteria. Of these, 16 patients were excluded due to incomplete data (*n* = 3), discharge during the study period (*n* = 10), or died before study endpoint (*n* = 3). Consequently, 302 patients were initially included in the study. Furthermore, an additional 8 patients were excluded owing to changes in the route of nutrition support (*n* = 5) or discontinuation of EN support (*n* = 3). Ultimately, data from 294 patients were included in the statistical analysis (Figure 1).

Table 1 presents the baseline characteristics, laboratory indices, and comparisons of patients who did and did not develop RFS on days 3 and 5 of EN. The mean age of the critically ill patients was 73.33 years, with 33.8% were female.

**TABLE 1:** Baseline characteristics and clinical characteristics of patients with and without RFS on the day 3 and 5 of EN.

Variables		Total	Day 3		*P*	Day 5		*P*
			No-RFS (*n* = 109)	RFS (*n* = 185)		No-RFS (*n* = 84)	RFS (*n* = 210)	
Gender, women *n* (%)		99 (33.7%)	36 (33.0%)	63 (34.1%)	0.857	26 (31.0%)	73 (34.8%)	0.532
Age, median (IQR), year		73.35 (66.04–84.04)	75.65 (68.02–85.04)	71.22 (65.51–83.02)	0.055	74.50 (68.01–83.82)	72.71 (66.02–84.04)	0.600
BMI median (IQR)		22.65 (20.85–24.23)	22.62 (20.52–24.20)	22.54 (20.81–24.25)	0.937	22.62 (20.55–24.34)	22.61 (20.80–24.24)	0.812
mNUTRIC (IQR)		5.78 (5.00–7.00)	5.86 (4.50–7.00)	5.73 (5.00–7.00)	0.487	5.87 (5.00–7.00)	5.74 (5.00–7.00)	0.068
APACHE II (IQR)		26.59 (18.00–32.00)	25.83 (16.00–31.00)	27.04 (18.00–32.00)	0.440	26.44 (16.00–32.00)	26.65 (18.00–30.00)	0.902
SOFA (IQR)		7.79 (5.00–9.00)	7.64 (5.00–9.50)	7.88 (5.00–9.00)	0.583	7.89 (5.00–10.00)	7.75 (5.00–9.00)	0.750
Admission diagnosis *n* (%)					0.115			0.073
	Respiratory	151 (54.4%)	64 (58.7%)	87 (47.0%)		52 (61.9%)	99 (47.1%)	
	Cardiovascular and circulatory	44 (15.0%)	11 (10.1%)	33 (17.8%)		10 (11.9%)	34 (16.2%)	
	Neurological	45 (15.3%)	18 (16.5%)	27 (14.6%)		6 (7.1%)	39 (18.6%)	
	Trauma	29 (9.9%)	10 (9.2%)	19 (10.3%)		8 (9.5%)	21 (10.0%)	
	Others	25 (8.5%)	6 (5.5%)	19 (10.3%)		8 (9.5%)	17 (8.1%)	
Comorbidities, *n* (%)					0.027			0.178
	0	82 (27.9%)	22 (20.2%)	60 (32.4%)		16 (19.0%)	66 (31.4%)	
	1	57 (19.4%)	25 (22.9%)	32 (17.3%)		20 (23.8%)	37 (17.6%)	
	2	68 (23.1%)	33 (30.3%)	35 (51.5%)		21 (25.0%)	47 (22.4%)	
	≥3	87 (29.6%)	29 (26.6%)	58 (31.4%)		27 (32.1%)	60 (28.6%)	
Baseline laboratory median (IQR)								
	Albumin	32.31 (28.42–35.15)	32.92 (28.52–35.54)	32.04 (28.42–34.85)	0.327	32.01 (28.82–34.23)	32.53 (28.25–36.12)	0.502
	Globulin	27.63 (23.41–31.02)	29.02 (24.85–32.70)	26.84 (23.02–29.90)	0.001	29.04 (24.25–32.92)	27.01 (23.41–30.02)	0.004
	Urea	14.22 (5.42–13.73)	12.72 (5.91–16.20)	15.21 (5.10–11.22)	0.001	14.12 (6.74–17.52)	14.31 (4.92–10.82)	<0.001
	Creatinine	94.02 (50.12–105.04)	100.15 (54.84–112.35)	90.42 (47.30–98.43)	0.098	105.72 (55.81–129.03)	89.35 (49.34–96.92)	0.053
	Uric acid	331.11 (207.72–420.52)	335.21 (220.84–434.15)	328.65 (202.24–409.03)	0.279	338.61 (197.42–434.31)	328.12 (213.53–416.54)	0.828
	Lactate dehydrogenase	350.72 (206.51–393.02)	344.72 (212.31–359.04)	354.21 (203.52–407.51)	0.801	374.92 (208.34–374.82)	340.74 (205.01–384.05)	0.860
	CK	360.82 (51.05–325.54)	219.01 (47.51–190.02)	445.43 (154.52–452.02)	0.001	278.8 (44.3–211.3)	394.43 (55.02–384.04)	0.010
	Hemoglobin	109.61 (89.05–127.30)	108.42 (90.51–127.52)	110.21 (89.02–127.52)	0.853	104.51 (83.02–124.02)	111.62 (91.85–130.05)	0.139
	Blood glucose	9.63 (6.83–11.05)	9.42 (6.51–11.12)	9.70 (6.82–10.92)	0.210	10.51 (7.23–12.02)	9.32 (6.61–10.40)	0.041
	Serum sodium	139.65 (136.42–142.43)	140.32 (137.34–143.14)	139.24 (136.35–142.23)	0.058	140.21 (136.80–143.02)	139.42 (136.41–142.23)	0.195
	Serum potassium	4.01 (3.61–4.32)	4.31 (3.83–4.63)	3.81 (3.52–4.23)	<0.001	4.40 (3.91–4.70)	3.81 (3.52–4.24)	<0.001
	Serum chlorine	104.44 (100.72–108.23)	104.90 (100.82–108.15)	104.10 (100.61–108.42)	0.444	104.75 (100.84–108.94)	104.31 (100.70–108.11)	0.516
	Serum magnesium	0.84 (0.75–0.91)	0.90 (0.82–1.03)	0.81 (0.72–0.92)	<0.001	0.93 (0.81–0.99)	0.80 (0.72–0.87)	<0.001
	Serum calcium	2.15 (2.05–2.24)	2.12 (2.03–2.24)	2.10 (1.91–2.23)	0.001	2.11 (2.02–2.21)	2.12 (2.03–2.24)	0.039
	Serum phosphorus	1.02 (0.81–1.22)	1.16 (0.93–1.31)	0.90 (0.70–1.10)	<0.001	1.22 (0.93–1.32)	0.91 (0.74–1.12)	<0.001
	CRP	70.22 (13.52–100.93)	60.84 (16.92–86.31)	75.72 (12.12–107.31)	0.291	71.20 (17.12–98.14)	69.73 (11.70–101.32)	0.783
LOS median (IQR)		12.51 (9.02–15.01)	12.82 (9.03–15.02)	12.32 (8.04–15.03)	0.231	12.31 (10.01–15.02)	12.62 (8.02–15.04)	0.329

BMI, body mass index; mNUTRIC, modified Nutrition Risk in the Critically ill; APACHE II, Acute Physiology and Chronic Health Evaluation II; SOFA, Sequential Organ Failure Assessment; CK, creatine kinase; CRP, C-reactive protein; IQR, interquartile range; LOS, ICU length of stay.

### 3.2 Incidence of RFS

In this study, 185 patients (62.9%) developed RFS on day 3 of EN, and 210 patients (71.4%) developed RFS on day 5 (*P* < 0.001) (Table 2, Supplementary Table 1).

**TABLE 2:** Intensive care unit (ICU) mortality according to RFS severity on days 3 and 5 of EN.

Time	RFS severity	Deaths/total, *n*/*N* (%)	Overall *P*	*P*	*P* for trend
Day 3			0.095		0.047
	No RFS	10/109 (9.2)		–	
	Mild RFS	5/55 (9.1)		1.000	
	Moderate/severe RFS	23/130 (17.7)		0.062	
Day 5			0.023		0.006
	No RFS	5/84 (6.0)		–	
	Mild RFS	9/81 (11.1)		0.273	
	Moderate/severe RFS	24/129 (18.6)		0.008	

On day 3 of EN, compared with patients who did not develop RFS, those who developed RFS had more comorbidities (*P* = 0.027), higher levels of urea (*P* = 0.001) and CK (*P* = 0.001), and lower levels of globulin, serum potassium, serum magnesium, serum calcium, and serum phosphate (*P* = 0.001, *P* < 0.001, *P* < 0.001, *P* = 0.001, *P* < 0.001, respectively).

On day 5 of EN, compared with patients who did not develop RFS, those who developed RFS had higher levels of urea (*P* < 0.001) and CK (*P* = 0.010), and lower levels of globulin, blood glucose, serum potassium, serum magnesium, serum calcium, and serum phosphate (*P* = 0.004, *P* = 0.041, *P* < 0.001, *P* < 0.001, *P* = 0.039, *P* < 0.001, respectively).

### 3.3 Clinical outcomes

Among the 294 patients, 38 (12.9%) died in ICU. ICU mortality increased with RFS severity at days 3 and 5 of EN, with the clearest gradient observed on day 5. On day 3, the overall difference among the no-RFS, mild-RFS, and moderate/severe-RFS groups was not significant (overall *P* = 0.095), although a significant upward trend was observed (*P* for trend = 0.047). On day 5, both the overall difference and the severity-related trend were significant (overall *P* = 0.023; *P* for trend = 0.006) (Table 2).

In further analyses, moderate/severe RFS was associated with significantly higher ICU mortality compared with the combined mild/no-RFS group on day 3 (OR = 2.14, 95% CI: 1.06–4.28, *P* = 0.036) and day 5 (OR = 2.47, 95% CI: 1.22–4.99, *P* = 0.014); however, direct comparisons between moderate/severe and mild RFS were not statistically significant at either time point (Supplementary Table 2).

### 3.4 Multivariate analysis of RFS

The results of the multivariable Firth penalized logistic regression analysis are presented in Table 3. On the day 3 of EN, patients with hypomagnesemia (OR: 8.85, 95% CI: 3.43–28.66, *P* < 0.001), hypokalemia (OR: 3.12, 95% CI: 1.38–7.68, *P* = 0.005), and hypophosphatemia (OR: 3.95, 95% CI: 1.94–863, *P* < 0.001) had higher incidence rate of RFS. Similarly, on the day 5 of EN, patients with hypomagnesemia (OR: 10.02, 95% CI: 3.22–49.59, *P* < 0.001), hypokalemia (OR: 6.75, 95% CI: 2.11–33.59, *P* < 0.001), and hypophosphatemia (OR: 3.87, 95% CI: 1.68–10.25, *P* = 0.001) had higher incidence rate of RFS. These findings indicate that low serum magnesium, potassium, and phosphorus levels were consistently associated with early RFS during EN support in critically ill patients. Although the association with hypomagnesemia remained statistically significant after Firth correction, its relatively wide confidence interval indicates limited precision in the estimated effect size. We also compared conventional multivariable logistic regression with Firth penalized logistic regression for factors associated with RFS on days 3 and 5 (Supplementary Table 3).

**TABLE 3:** Multivariable firth penalized logistic regression analysis.

Variable	RFS-day 3			RFS-day 5		
	RFS, *n*/*N* (%)	Firth-adjusted OR (95% CI)	*P*	RFS, *n*/*N* (%)	Firth-adjusted OR (95% CI)	*P*
Serum magnesium						
0.75–1.25 (reference)	118/219 (53.9)			144/219 (65.8)		
<0.75	64/68 (94.1)	8.85 (3.43–28.66)	<0.001	65/68 (95.6)	10.02 (3.22–49.59)	<0.001
>1.25	3/7 (42.9)	1.00 (0.14–7.97)	0.996	1/7 (14.3)	0.081 (0.003–1.036)	0.005
Serum potassium						
3.50–5.50 (reference)	129/223 (57.8)			150/223 (67.3)		
<3.50	53/61 (86.9)	3.12 (1.38–7.68)	0.005	59/61 (96.7)	6.75 (2.11–33.59)	<0.001
>5.50	3/10 (30.0)	0.47 (0.07–2.29)	0.360	1/10 (10.0)	0.070 (0.005–0.481)	0.005
Serum phosphorus						
0.81–1.45 (reference)	109/194 (56.2)			131/194 (67.5)		
<0.81	73/84 (86.9)	3.95 (1.94–8.63)	<0.001	77/84 (91.7)	3.87 (1.68–10.25)	0.001
>1.45	3/16 (18.8)	0.52 (0.11–1.87)	0.330	2/16 (12.5)	0.30 (0.054–1.16)	0.083

## 4 Discussion

### 4.1 Overview of key findings

In this prospective observational study, we found that more than half of critically ill patients receiving EN experienced RFS. The incidence of RFS reached 62.9% by day 3 and further increased to 71.4% by day 5 (*P* < 0.001). After stratifying patients by RFS severity, the mortality risk was primarily concentrated in patients with moderate/severe RFS, suggesting that persistent or worsening RFS may be associated with poorer clinical outcomes. In contrast, the mortality difference between patients with mild RFS and those without RFS was not significant. Multivariate analysis demonstrated that baseline serum magnesium, phosphorus, and potassium levels were independent risk factors for predicting early RFS during EN. These findings underscore that the early stage of nutritional support represents a high-risk window for this complication and highlight the importance of systematic electrolyte monitoring in critically ill patients.

### 4.2 Incidence of RFS

A retrospective study employing the NICE and ASPEN criteria demonstrated that the incidence of RFS in medically critically ill patients was 22.7% and 27.3%, respectively, with no statistically significant difference in 30-days mortality between the two groups (37.3% vs. 33.8%, *P* = 0.627) (24). Another study reported an RFS incidence of up to 50.0% in ICU patients (14). The higher RFS incidence observed in our study may be significantly associated with the age distribution of our population (≥60 years old accounted for 84.7%). Critically ill patients often present with underlying malnutrition due to hypermetabolic states and disease-related catabolism, while elderly patients face additional risk factors such as physiological decline, prolonged bed rest, and mechanical ventilation, further exacerbating their susceptibility to RFS.

Friedli et al. (1) found that RFS predominantly occurs within the 72 h following the initiation of nutritional support. Patients with RFS showed significantly higher in-hospital mortality rates and prolonged length of stay, findings consistent with those reported by Lara et al. (6). Friedli et al. (5) further demonstrated that 36.5% of medical patients exhibited RFS-related electrolyte disturbances or clinical symptoms, with a 180-days mortality rate of 29.8% in RFS patients compared with 17.5% in non-RFS patients (*P* < 0.050). Ferlicolak et al. reported a 30% incidence of refeeding hypophosphatemia in critically ill patients aged ≥ 80 years, lower than the RFS incidence observed in our cohort. This difference may reflect variations in diagnostic criteria and assessment windows, as their study focused on post-feeding hypophosphatemia, whereas ours applied the ASPEN criteria based on changes in phosphorus, potassium, and magnesium within 5 days of nutritional support (25). Moreover, the 180-days mortality rate in RFS patients was 29.8%, significantly higher than 17.5% of the non-RFS patients (*P* < 0.050). At the same time, the risk of RFS patients being transferred to the ICU was significantly increased. Multiple studies have confirmed a significant association between RFS risk stratification and patient prognosis. The 30-days mortality rate was 5.0% in patients without RFS risk, compared with 16.3% in the high-risk group (16). A prospective observational study in Germany confirmed that early risk assessment and dynamic monitoring of high-risk RFS patients can significantly reduce the incidence of RFS-related adverse (26).

Currently, diagnostic criteria for RFS remains incompletely standardized. The ASPEN definition relies on electrolyte abnormalities, whereas a more clinically applicable framework should also consider nutritional risk, feeding exposure, and new-onset organ dysfunction related to refeeding. In this study, nutritional exposure and the timing of clinical manifestations were not systematically recorded. Therefore, some identified cases may have reflected biochemical abnormalities without overt organ dysfunction, potentially contributing to the high observed incidence. Despite this limitation, existing evidence consistently indicates that RFS is a common and clinically significant complication of EN in critically ill patients. Future prospective studies should integrate nutritional exposure, electrolyte changes, and clinical manifestations to improve diagnostic accuracy and clinical applicability (3, 27).

### 4.3 Risk factors for RFS

This study further explored risk factors associated with the early occurrence of RFS in critically ill patients. Multivariate analysis demonstrated that abnormal serum magnesium, phosphorus, and potassium levels at ICU admission were independently associated with the occurrence of RFS during EN support. The association between low serum magnesium and RFS remained significant after Firth penalized logistic regression was applied to reduce small-sample bias. However, the wide confidence interval indicated limited precision, and the magnitude of this association should therefore be interpreted cautiously. These findings are consistent with the results of several previous studies (13, 28). Studies by Adika et al. confirmed that baseline levels of phosphorus, magnesium, and potassium were significantly correlated with the incidence of severe RFS, with baseline potassium identified as the optimal predictor of RFS occurrence (29). Additionally, research on children and adolescents with anorexia nervosa showed that, according to the ASPEN diagnostic criteria, 41.0% of cases developed RFS (87.0% mild, 6.5% moderate, and 6.5% severe). Laboratory tests revealed hypophosphatemia in 50.0% of patients, hypokalemia in 48.0%, and hypomagnesemia in 30.0%, with RFS primarily manifesting as hypophosphatemia in 20.0% of cases (30). These findings strongly support the close association between electrolyte disturbances and the development of RFS (31). First, baseline electrolyte levels represent the pre-existing metabolic reserve capacity of the patient, which is conceptually distinct from the dynamic electrolyte depletion that defines RFS. Second, the pathophysiological mechanisms underlying baseline electrolyte abnormalities differ fundamentally from those driving the acute intracellular shift induced by refeeding. In clinical practice, baseline hypophosphatemia in critically ill patients is typically attributable to chronic conditions such as prolonged malnutrition, alcohol abuse, or chronic diuretic use. These factors deplete intracellular phosphate stores prior to EN initiation and operate independently of nutritional support, rather than being directly caused by EN itself. Meanwhile, studies have shown that older age was associated with higher odds of developing low phosphorus and low magnesium (32). Based on the above research evidence, the diagnostic criteria for RFS incorporate electrolyte indicators with sufficient scientific basis. Although serum phosphorus remains the most widely accepted diagnostic indicator for RFS, there is still academic controversy regarding the clinical value of other electrolyte indicators. This study confirmed that serum phosphorus, potassium, and magnesium levels are all significantly associated with RFS and can serve as predictive factors for the occurrence of RFS. These findings provide an evidence-based support for future revisions of clinical diagnostic criteria.

Although electrolyte derangements are clearly associated with the development of RFS, dynamic electrolyte monitoring remains inadequate in clinical practice. A prospective observational study conducted in the emergency department revealed that electrolyte assessments were not systematically performed in all patients receiving EN who were at risk of RFS. Another study also highlighted deficiencies in risk screening awareness, with data showing hypophosphatemia in 40.0%, hypomagnesemia in 25.0%, and hypokalemia in 15.7% of at-risk patients (31). The ASPEN explicitly recommends in its RFS consensus statement that patient at risk should receive prophylactic thiamine and multivitamin supplementation, along with standardized electrolyte monitoring protocols (30). Additionally, continuous assessment of RFS risk during nutritional support therapy and timely interventions are emphasized (32). The ASPEN also emphasizes the efficacy of integrating early detection and prevention strategies for RFS into malnutrition treatment protocols for mitigating severe outcomes (22). However, knowledge gaps remain a major barrier. Current data indicate that only about 18.0% of critical care physicians can correctly identify and manage RFS (31). A German cross-sectional study further confirmed that only 14.0% of clinicians were capable of diagnosing RFS (21). This lack of clinical awareness directly contributes to insufficient attention to RFS in practice, resulting in persistently high incidence rates and significant underdiagnosis. Compared with subjective, non-specific clinical manifestations, electrolyte levels serve as objective, quantifiable biomarkers that are more suitable for early identification and risk stratification. In the critical-care context, patients often lack typical symptoms due to mechanical ventilation, sedation, analgesia, or underlying diseases, making clinical presentations easily masked or misinterpreted. Consequently, dynamic monitoring of electrolyte concentrations is of paramount importance for early detection and risk prediction of RFS in critically ill patients. Guided by these considerations, the present study adopted the diagnostic criteria established by the ASPEN to maximize scientific rigor and ensure reproducibility of the findings.

### 4.4 Limitations

Despite these findings, several limitations of this study should be acknowledged. First, as a prospective observational study, we observed the occurrence of RFS within the first 5 days of EN support, lacking long-term follow-up data and systematic electrolyte dynamic monitoring. In addition, actual nutritional delivery and electrolyte supplementation were not systematically recorded. Therefore, we could not accurately assess the effects of nutritional exposure and reactive electrolyte replacement on the occurrence and severity of RFS. Future prospective studies should extend the observation period and systematically document nutritional delivery or electrolyte supplementation parameters to better evaluate their relationships with RFS occurrence, severity, and clinical outcomes. Second, only a single diagnostic criterion for RFS was applied; the absence of comparative analyses with alternative definitions may limit the external validity of our results. At last, the concurrent role of baseline electrolytes as both predictors and diagnostic criteria constituents introduces a risk of methodological circularity, which may overestimate their predictive value. Future studies are warranted to develop and validate non-circular prediction models based exclusively on pre-EN clinical variables to corroborate the findings of the present study.

## 5 Conclusion

Our prospective findings demonstrated that RFS incidence remains high among critically ill patients receiving EN, with a progressive increase during the early phase. Patients who developed moderate/severe RFS on days 3 and 5 had significantly higher ICU mortality than those mild/no-RFS patients. Baseline serum phosphorus, potassium, and magnesium at admission were risk factors for RFS. These results highlight the need for dynamic electrolyte monitoring through interdisciplinary collaboration to enable early RFS risk assessment.

## Data Availability

The datasets presented in this article are not readily available due to privacy and ethical restrictions. Requests to access the datasets should be directed to the the corresponding author.

## References

[1] Friedli N Stanga Z Sobotka L Culkin A Kondrup J Laviano A et al. Revisiting the refeeding syndrome: results of a systematic review. *Nutrition*. (2017) 35:151–60. 10.1016/j.nut.2016.05.016 28087222

[2] Preiser JC van Zanten AR Berger MM Biolo G Casaer MP Doig GS et al. Metabolic and nutritional support of critically ill patients: consensus and controversies. *Crit Care*. (2015) 19:35. 10.1186/s13054-015-0737-8 25886997 PMC4310041

[3] Reber E Friedli N Vasiloglou MF Schuetz P Stanga Z. Management of refeeding syndrome in medical inpatients. *J Clin Med*. (2019) 8:2202. 10.3390/jcm8122202 31847205 PMC6947262

[4] Singer P Blaser AR Berger MM Alhazzani W Calder PC Casaer MP et al. ESPEN guideline on clinical nutrition in the intensive care unit. *Clin Nutr*. (2019) 38:48–79. 10.1016/j.clnu.2018.08.037 30348463

[5] Zeki S Culkin A Gabe SM Nightingale JM. Refeeding hypophosphataemia is more common in enteral than parenteral feeding in adult in patients. *Clin Nutr*. (2011) 30:365–8. 10.1016/j.clnu.2010.12.001 21256638

[6] Zhang W Zhang SX Chen SF Yu T Tang Y. Development and validation of risk prediction model for refeeding syndrome in neurocritical patients. *Front Nutr.* (2023) 10:1083483. 10.3389/fnut.2023.1083483 36875840 PMC9975392

[7] Doig GS Heighes PT Simpson F Sweetman EA Davies AR. Early enteral nutrition, provided within 24 h of injury or intensive care unit admission, significantly reduces mortality in critically ill patients: a meta-analysis of randomised controlled trials. *Intensive Care Med*. (2009) 35:2018–27. 10.1007/s00134-009-1664-4 19777207

[8] Amadi B Besa E Zyambo K Kaonga P Louis-Auguste J Chandwe K et al. Impaired barrier function and autoantibody generation in malnutrition enteropathy in Zambia. *EBioMedicine*. (2017) 22:191–9. 10.1016/j.ebiom.2017.07.017 28750860 PMC5552244

[9] Meyer R Arpe L Kansu A Kelly V Lindley K O’Meara M et al. Gastrointestinal changes in paediatric malnutrition that may impact on nutrition choice. *Front Pediatr*. (2025) 13:1523613. 10.3389/fped.2025.1523613 40129696 PMC11931439

[10] Schneeweiss-Gleixner M Haselwanter P Schneeweiss B Zauner C Riedl-Wewalka M. Hypophosphatemia after start of medical nutrition therapy indicates early refeeding syndrome and increased electrolyte requirements in critically ill patients but has no impact on short-term survival. *Nutrients*. (2024) 16:922. 10.3390/nu16070922 38612956 PMC11013904

[11] Md Ralib A Mat Nor MB. Refeeding hypophosphataemia after enteral nutrition in a Malaysian intensive care unit: risk factors and outcome. *Asia Pac J Clin Nutr.* (2018) 27:329–35. 10.6133/apjcn.062017.09 29384319

[12] Yoshida M Suzuki M Wakatake H Kurisu M Saito H Ohshima Y et al. Association between poor outcomes and risk of refeeding syndrome among patients urgently admitted to the high dependency unit: a single-center cohort study in Japan. *Nutrients*. (2024) 16:3287. 10.3390/nu16193287 39408254 PMC11478408

[13] Yoshida M Izawa J Wakatake H Saito H Kawabata C Matsushima S et al. Mortality associated with new risk classification of developing refeeding syndrome in critically ill patients: a cohort study. *Clin Nutr*. (2021) 40:1207–13. 10.1016/j.clnu.2020.07.034 32828568

[14] Aubry E Friedli N Schuetz P Stanga Z. Refeeding syndrome in the frail elderly population: prevention, diagnosis and management. *Clin Exp Gastroenterol*. (2018) 11:255–64. 10.2147/ceg.S136429 30022846 PMC6045900

[15] Olsen SU Tazmini K Aas AM Ranhoff AH Pripp AH Hesseberg K et al. The incidence and mortality of refeeding syndrome in older hospitalized patients, based on three different diagnostic criteria: a longitudinal study. *Clin Nutr ESPEN*. (2024) 61:101–7. 10.1016/j.clnesp.2024.03.006 38777421

[16] Cioffi I Ponzo V Pellegrini M Evangelista A Bioletto F Ciccone G et al. The incidence of the refeeding syndrome. A systematic review and meta-analyses of literature. *Clin Nutr*. (2021) 40:3688–701. 10.1016/j.clnu.2021.04.023 34134001

[17] Naik NM Li J Seres D Freedberg DE. Assessment of refeeding syndrome definitions and 30-day mortality in critically ill adults: a comparison study. *JPEN J Parenter Enteral Nutr*. (2023) 47:993–1002. 10.1002/jpen.2560 37689982

[18] Friedli N Stanga Z Culkin A Crook M Laviano A Sobotka L et al. Management and prevention of refeeding syndrome in medical inpatients: an evidence-based and consensus-supported algorithm. *Nutrition*. (2018) 47:13–20. 10.1016/j.nut.2017.09.007 29429529

[19] Silva JSV Seres DS Sabino K Adams SC Berdahl GJ Citty SW et al. ASPEN consensus recommendations for refeeding syndrome. *Nutr Clin Pract*. (2020) 35:178–95. 10.1002/ncp.10474 32115791

[20] Doig GS Simpson F Heighes PT Bellomo R Chesher D Caterson ID et al. Restricted versus continued standard caloric intake during the management of refeeding syndrome in critically ill adults: a randomised, parallel-group, multicentre, single-blind controlled trial. *Lancet Respir Med*. (2015) 3:943–52. 10.1016/s2213-2600(15)00418-x 26597128

[21] Janssen G Pourhassan M Lenzen-Großimlinghaus R Jäger M Schäfer R Spamer C et al. The refeeding syndrome revisited: you can only diagnose what you know. *Eur J Clin Nutr*. (2019) 73:1458–63. 10.1038/s41430-019-0441-x 31127188

[22] Terlisten K Wirth R Daubert D Pourhassan M. Refeeding syndrome in older hospitalized patients: incidence, management, and outcomes. *Nutrients*. (2023) 15:4084. 10.3390/nu15184084 37764866 PMC10535909

[23] Tongyoo S Rawangban P Naorungroj T. Prevalence, predictive factors, and outcomes of refeeding syndrome among medically critically ill patients: a retrospective cohort study. *Nutr Clin Pract*. (2025) 40:125–33. 10.1002/ncp.11160 38864503

[24] Rio A Whelan K Goff L Reidlinger DP Smeeton N. Occurrence of refeeding syndrome in adults started on artificial nutrition support: prospective cohort study. *BMJ Open*. (2013) 3:e002173. 10.1136/bmjopen-2012-002173 23315514 PMC3549252

[25] Ferlicolak L Altintas ND. Refeeding hypophosphatemia in oldest old critically ill patients. *Ir J Med Sci*. (2024) 193:1085–9. 10.1007/s11845-023-03498-0 37589868

[26] Adika E Jia R Li J Seres D Freedberg DE. Evaluation of the ASPEN guidelines for refeeding syndrome among hospitalized patients receiving enteral nutrition: a retrospective cohort study. *JPEN J Parenter Enteral Nutr*. (2022) 46:1859–66. 10.1002/jpen.2368 35274317 PMC9464262

[27] Pruccoli J Barbieri E Visconti C Pranzetti B Pettenuzzo I Moscano F et al. Refeeding syndrome and psychopharmacological interventions in children and adolescents with anorexia nervosa: a focus on olanzapine-related modifications of electrolyte balance. *Eur J Pediatr*. (2024) 183:1935–41. 10.1007/s00431-024-05430-9 38347260 PMC11001716

[28] Pérsico RS Franzosi OS. Patients with enteral nutrition at risk of refeeding syndrome show electrolyte abnormalities at admission in the emergency department. *Nutr Hosp*. (2021) 38:897–902. 10.20960/nh.03500 34148348

[29] Nagata JM Nguyen A Vargas R Downey AE Chaphekar AV Ganson KT et al. Sex differences in electrolyte abnormalities indicating refeeding syndrome risk among hospitalized adolescents and young adults with eating disorders. *J Eat Disord*. (2024) 12:67. 10.1186/s40337-024-01012-0 38790035 PMC11127403

[30] Matthews-Rensch K Blackwood K Lawlis D Breik L McLean C Nguyen T et al. The Australasian society of parenteral and enteral nutrition: consensus statements on refeeding syndrome. *Nutr Diet*. (2025) 82:128–42. 10.1111/1747-0080.70003 40090863 PMC11973624

[31] Bahashwan SM Sindy AA Azzeh F Alkholy SO Abusudah WF Bukhari HM et al. Refeeding syndrome awareness among physicians of king abdullah medical city in Makkah, Saudi Arabia. *Healthcare*. (2023) 11:794. 10.3390/healthcare11060794 36981452 PMC10048025

[32] Friedli N Odermatt J Reber E Schuetz P Stanga Z. Refeeding syndrome: update and clinical advice for prevention, diagnosis and treatment. *Curr Opin Gastroenterol*. (2020) 36:136–40. 10.1097/mog.0000000000000605 31895231

