# Supplementary material for: Incidence, risk factors and outcomes of refeeding syndrome in critically ill patients receiving enteral nutrition: a prospective observational study
Source: Front Med (Lausanne). 2026 Aug 31;13:1923682. doi: 10.3389/fmed.2026.1923682 (PMC13572628; doi:10.3389/fmed.2026.1923682)
Supplement: Supplementary file 1 [file Table_1.docx]

Supplementary_Tables_S1-S3

Table S1 Distribution and mortality according to RFS severity on Days 3 and 5

| **RFS severity** | | **Day 3** | | | **Day 5** | | |
| --- | --- | --- | --- | --- | --- | --- | --- |
|  |  | n | % | Mortality, % | n | % | Mortality, % |
| RFS |  |  |  |  |  |  |  |
|  | none | 109 | 37.1 | 9.2 | 84 | 28.6 | 6.0 |
|  | mild | 55 | 18.7 | 9.1 | 81 | 27.6 | 11.1 |
|  | moderate | 41 | 13.9 | 14.6 | 76 | 25.9 | 19.7 |
|  | severe | 89 | 30.3 | 19.1 | 53 | 18.0 | 17.0 |

Table S2 Comparisons of mortality according to RFS severity on Days 3 and 5

| **Time** | **Comparsion** | ***P*** |
| --- | --- | --- |
| Day 3 |  |  |
|  | Mild RFS vs no RFS | 1.000 |
|  | Moderate/severe RFS vs no RFS | 0.062 |
|  | Moderate/severe RFS vs mild RFS | 0.179 |
|  | Moderate/severe RFS vs mild RFS/no RFS | 0.036 |
| Day 5 |  |  |
|  | Mild RFS vs no RFS | 0.273 |
|  | Moderate/severe RFS vs no RFS | 0.008 |
|  | Moderate/severe RFS vs mild RFS | 0.175 |
|  | Moderate/severe RFS vs mild RFS/no RFS | 0.014 |

Table S3 Comparison of Multivariate logistic and Firth penalized logistic regression analyses for factors associated with RFS on Days 3 and 5

| **Variables** | **Day 3** | | | | **Day 5** | | | |
| --- | --- | --- | --- | --- | --- | --- | --- | --- |
|  | **logistic regression** | | **Firth logistic regression** | | **logistic regression** | | **Firth logistic regression** | |
|  | ***OR(95%CI)*** | ***p*** | ***OR (95% CI)*** | ***P*** | ***OR(95%CI)*** | ***p*** | ***OR (95% CI)*** | ***P*** |
| Serum magnesium |  |  |  |  |  |  |  |  |
| 0.75-1.25（reference） |  |  |  |  |  |  |  |  |
| ＜0.75 | 11.11(3.76-32.83) | ＜.001 | 8.85 (3.43–28.66) | ＜0.001 | 12.40(2.83-54.31) | 0.001 | 10.02 (3.22–49.59) | ＜0.001 |
| ＞1.25 | 1.31(0.17-10.37) | 0.800 | 1.00 (0.14–7.97) | 0.996 | 0.04(0.00-0.97) | 0.048 | 0.081 (0.003–1.036) | 0.005 |
| Serum potassium |  |  |  |  |  |  |  |  |
| 3.50-5.50（reference） |  |  |  |  |  |  |  |  |
| ＜3.50 | 3.34(1.43-7.80) | 0.005 | 3.12 (1.38–7.68) | 0.005 | 11.00(2.41-50.15) | 0.002 | 6.75 (2.11–33.59) | ＜0.001 |
| ＞5.50 | 0.43(0.08-2.30) | 0.323 | 0.47 (0.07–2.29) | 0.360 | 0.04(0.00-0.52) | 0.014 | 0.070 (0.005–0.481) | 0.005 |
| Serum phosphorus |  |  |  |  |  |  |  |  |
| 0.81-1.45（reference） |  |  |  |  |  |  |  |  |
| ＜0.81 | 4.29(2.05-8.95) | ＜ 0.001 | 3.95 (1.94–8.63) | ＜0.001 | 1.54(1.80-11.4) | 0.001 | 3.87 (1.68–10.25) | 0.001 |
| ＞1.45 | 0.34(0.08-1.38) | 0.131 | 0.52 (0.11–1.87) | 0.330 | 0.19(0.40-0.90) | 0.037 | 0.30 (0.054–1.16) | 0.083 |
